# Cross-kingdom regulation of plant microRNAs: potential application in crop improvement and human disease therapeutics

**DOI:** 10.3389/fpls.2024.1512047

**Published:** 2024-12-17

**Authors:** Lei Shi, Chao Guo, Miaomiao Fang, Yingmei Yang, Fei Yin, Yuan Shen

**Affiliations:** ^1^ International Joint Research Laboratory for Recombinant Pharmaceutical Protein Expression System of Henan, Xinxiang Medical University, Xinxiang, China; ^2^ School of Basic Medical Sciences, Xinxiang Medical University, Xinxiang, China; ^3^ School of Pharmacy, Xinxiang Medical University, Xinxiang, China; ^4^ National Demonstration Center for Experimental (Aquaculture) Education, School of Marine Sciences, Ningbo University, Ningbo, China

**Keywords:** plant miRNAs, miRNA biosynthesis, cross-kingdom regulation, miRNA transfer, plant-derived exosome-like nanoparticles

## Abstract

Plant microRNAs (miRNAs) are small non-coding RNA molecules that usually negatively regulate gene expression at the post-transcriptional level. Recent data reveal that plant miRNAs are not limited to individual plants but can transfer across different species, allowing for communication with the plant, animal, and microbial worlds in a cross-kingdom approach. This review discusses the differences in miRNA biosynthesis between plants and animals and summarizes the current research on the cross-species regulatory effects of plant miRNAs on nearby plants, pathogenic fungi, and insects, which can be applied to crop disease and pest resistance. In particular, this review highlights the latest findings regarding the function of plant miRNAs in the transboundary regulation of human gene expression, which may greatly expand the clinical applicability of plant miRNAs as intriguing tools in natural plant-based medicinal products in the future.

## 1 Introduction

MicroRNAs (miRNAs), a category of non-coding RNAs, share a conserved single-stranded RNA structure with a length between 18 and 24 nucleotides. These miRNAs regulate target gene expression in eukaryotes at the post-transcriptional level. The first miRNA was identified as lin-4 in *Caenorhabditis elegans*. Since then, miRNAs have been found in an ever-expanding family across animals, plants, and other eukaryotic species. Plant miRNAs were initially identified in *Arabidopsis thaliana* (Llave et al., 2002). Subsequently, miRNAs in other plant species have been identified one after another. Due to advances in next-generation sequencing (NGS) technology, tens of thousands of miRNAs have been discovered in various plants. For instance, 10,414 mature miRNA sequences and 8,615 hairpin precursors from 82 plant organisms have been reported present in the most recent version of miRbase (miRbase v22, https://mirbase.org/index.shtml) (Kozomara et al., 2019). Approximately 16,438 miRNA sequences from 121 plant species have been cataloged in the plant non-coding RNA database (PNRD, http://structuralbiology.cau.edu.cn/PNRD/index.php) (Yi et al., 2015). Additionally, about 38,186 miRNA sequences from 179 plants have been documented in the second version of Plant miRNA database (PmiREN v2.0, https://pmiren.com/) (Guo et al., 2022). Current understanding of plant miRNA function mainly focuses on regulating the expression of target genes during plant development and in response to stress. However, mounting evidence suggests that plant miRNAs not only function within plant bodies but also cross-regulate the expression of genes in viruses, fungi, insects, and mammals (Wang et al., 2017c; Chen and Rechavi, 2022; Sun et al., 2022; Wang et al., 2022; Li et al., 2023). This paper reviews recent research progress on the transfer mechanisms of plant miRNAs and cross-kingdom miRNA/target gene regulation systems. In particular, examples of cross-boundary transmission and regulation of plant miRNAs, along with their possible target genes, are summarized here, along with their potential applications in developing new drugs.

## 2 Biosynthesis of miRNAs

**Figure 1:**
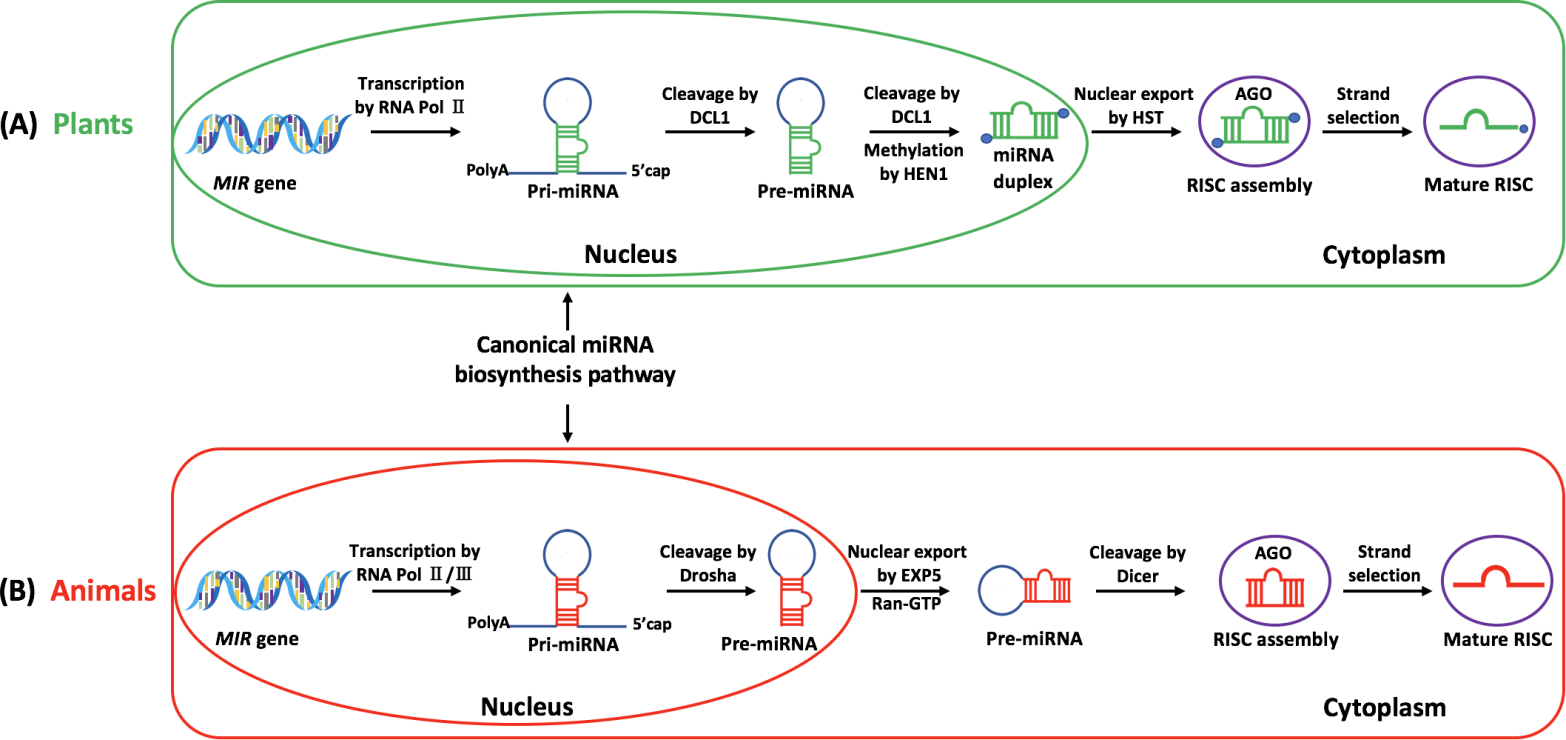
The biosynthesis and maturation of miRNAs in plants **(A)** and animals **(B)**.

MiRNAs are produced through several steps, including transcription of miRNA encoding genes (*MIR* genes), primary miRNA (pri-miRNA) processing, and mature miRNA export (Ameres and Zamore, 2013). The processes of miRNA biosynthesis in both plants and animals are illustrated in **Figure 1**. In plants, the pri-miRNAs are mostly synthesized by RNA polymerase II (Pol II). Then, the DICER-LIKE 1 (DCL1) protein processes pri-miRNAs, removing the poly-A tail and producing short precursors known as pre-miRNAs. DCL1 further processes the secondary structure of pre-miRNAs to produce the imperfect miRNA-miRNA* duplexes. Consequently, the miRNA-miRNA* duplexes are methylated at the 3’ terminal by HUA enhancer 1 (HEN1) in plants and then transported from nucleus to cytoplasm, associated with HASTY (HST). Finally, the duplexes separate, and only one strand is integrated into the RNA-induced silencing complex (RISC) to identify target mRNAs (Yu et al., 2017). In animals, the *MIR* genes are transcribed as pri-miRNAs by RNA polymerase II and III (Pol II/III). Then RNase III enzyme Drosha cleaves the poly-A tails from pri-miRNAs in the nucleus to produce pre-miRNAs with a 2 nt overhang at the 3’ end and a 5’ phosphate. The pre-miRNAs are subsequently processed and transferred to the cytoplasm concurrently by Ran-GTP dependent EXPORTIN 5 (EXP5). Dicer finally cleaves the pre-miRNAs to produce ~22 bp mature miRNA duplexes, which combine with ARGONAUTE (AGO) proteins to form RISC (**Figure 1**).

Based on the diversity in biosynthesis processes, there are several differences between animal miRNAs and plant miRNAs: (1) Compared to animal miRNAs, plant miRNAs have longer and more varied stem-loop precursors (ranging from 100 to 900 nt in plants *vs.* predominantly less than 100 nt in animals) (Zhu et al., 2016). (2) The length of plant miRNAs shows a tighter distribution around 21 nt (Cao et al., 2023), while animal miRNAs typically range from 22 to 23 nt (Ha and Kim, 2014). Interestingly, abnormal miRNA isoforms with 1-nt-shorter 3’ends are widely accumulated in many human primary tumors and ectopic expression of plant RNA-dependent RNA polymerase 1 (RDR1) enables broad antitumor response by rescuing miRNA deficiency (Qi et al., 2022). (3) At the 5’ terminus, plant miRNAs tend to favor uracil (U). Additionally, the 3’ terminal ribose of plant miRNAs is methylated at the 2′-hydroxyl group by HEN1, providing molecular stability, which is a crucial step for miRNA biosynthesis in plants but not in animals (Baranauske et al., 2015). (4) Animals and plants exhibit distinct miRNA genetic structures. About 50% of genes encoding animal miRNAs are located in clusters, which frequently comprise different mature miRNAs. For example, one of the most deeply conserved clusters is the mir-100~let-7~mir-125 cluster, which has an important role in the development of bilaterian animals (Roush and Slack, 2008). In contrast, there are fewer instances of miRNA clusters in plants, which almost exclusively encode miRNAs that share high homology. An example of high homology of plant miRNAs is the *miR159/miR319* family in *Arabidopsis*, which share identical sequence at 17 of 21 nucleotides, yet evolve to target two distinct gene families (Palatnik et al., 2007). (5) Differences can also be found in miRNA target recognition and location, as well as their mode of action. In animals, miRNA complementary sites are often found in the 3’ untranslated regions (3’ UTR), whereas in most plants, they are nearly exclusively present within the open reading frame regions. Plant miRNAs usually have single binding sites, and exhibit high or perfect complementarity to their target sites (less than 4 mismatches), whereas animal miRNAs can bind to multiple target sites with less complementarity. Moreover, plant miRNAs primarily repress gene expression by directly cleaving target mRNAs at the post-transcriptional level (Rogers and Chen, 2013), whereas animal miRNAs mainly repress the translation of target genes by preventing translational initiation or elongation. Despite differences in target recognition, plant miRNAs can also induce translational repression in addition to target cleavage (Gandikota et al., 2007; Iwakawa and Tomari, 2015). Notably, the miRNA regulatory mechanisms of gene expression are complicated and vary in different organisms and different tissues (Fabian et al., 2010). In some situations, miRNAs can also increase mRNA stability or translation efficiency (Henke et al., 2008; Orom et al., 2008). For instance, mammalian miR-10a interacts with 5’ UTR of ribosomal protein mRNAs and enhances their translation in response to stress or nutrient shortage (Orom et al., 2008). MiR-122 enhances the replication of the hepatitis C virus (HCV) by binding to 5’UTR and stabilizing the RNA of HCV (Jopling et al., 2005; Henke et al., 2008).

## 3 The roles of plant miRNAs in cross-boundary regulation

The cross-kingdom regulation by plant miRNAs was first reported in 2012 when it was discovered that rice-derived miR168a existed in mouse sera and functioned in the liver by targeting low-density lipoprotein receptor adapter protein 1 (LDLRAP1) (Zhang et al., 2012). Notably, some plant miRNAs are highly homologous to animal miRNAs (Bellato et al., 2019; Avsar et al., 2020; Cao et al., 2023; Huang et al., 2023), and many biological processes mediated by miRNAs are shared between plants and other organisms (Arda and Doganlar, 2022), therefore facilitating plant miRNAs’ capacity for cross-kingdom modulation of gene expression (Xu et al., 2024b). Recent research has demonstrated that small RNAs, especially miRNAs, can move not only within the cells and tissues of a single plant but also between distinct species, enabling cross-kingdom communication (Knip et al., 2014; Bellato et al., 2019; Zeng et al., 2019; Gualtieri et al., 2020; Chen and Rechavi, 2022; Urzi et al., 2022). The trans-species transfer and regulation of miRNAs have been documented between plants and other organisms, including plants-plants, plants-viruses, plants-fungi, plants-insects, plants-animals, and plants-humans (Zeng et al., 2019; Zhang et al., 2019; Chi et al., 2023; Li et al., 2023). Several recent findings regarding the role of plant miRNAs in mediating cross-species gene regulation are reviewed as follows. Additionally, this review provides a mechanistic understanding of miRNA regulation, including plant miRNA transfer carriers, plant miRNA absorption, plant miRNA activity at the destination, and the future application of plant miRNAs.

### 3.1 The miRNA communication between plants

**Figure 2:**
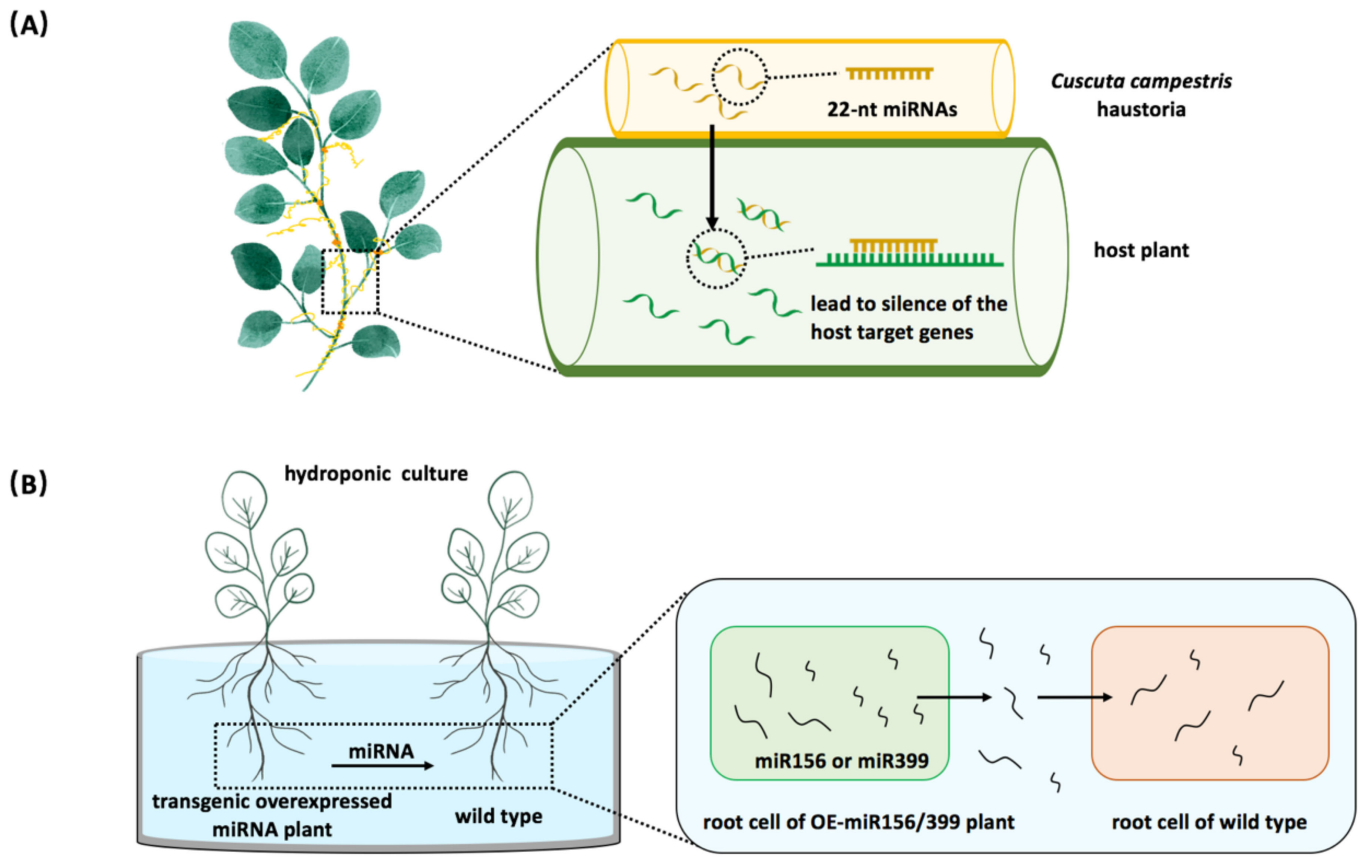
Plant miRNAs serve as regulatory molecules between plants. **(A)** The inter-species miRNA regulation occurs between the parasitic plant dodder (*Cuscuta campestris*) and its host. *Cuscuta campestris* utilizes haustoria to facilitate the movement of miRNAs into host plant cells. **(B)** Plant miRNAs act as signaling molecules affecting nearby plants. When wild-type plants are co-cultivated with overexpressing miR156 or miR399 plants, the expression levels of miRNA target genes are decreased in wild-type plants.

It has been reported that trans-species miRNA communication occurs naturally between the parasitic and host plants (Shahid et al., 2018). *Cuscuta campestris* obtains nutrients from host plants by using the feeding structure known as ‘haustoria’. In the haustoria, *C. campestris* generates 22-nt miRNAs that are also found in the stems of its host plants (**Figure 2A**) (Shahid et al., 2018). These parasitic plant 22-nt miRNAs function in host plants, causing the host target genes to be silenced, suggesting a natural inter-species miRNA regulatory relationship between the parasitic plant and its host (Shahid et al., 2018). Recent studies have confirmed that plant miRNAs can be secreted and taken up by the nearby plants (Betti et al., 2021; Marzec, 2022). *Arabidopsis* miR399 and miR156 are shown to target *PHO2* and *SPL* genes, respectively (Pant et al., 2008; Bhogale et al., 2014). Exogenous administration of chemically synthesized miR399 or miR156 has been shown to trigger the silencing of their target genes in plants (Betti et al., 2021). The observed silencing effect was even more dramatic when endogenous miRNA extracts from plants overexpressing miR399 or miR156 were used instead of synthetic products, demonstrating that miRNAs can be absorbed and modulate target gene expression in receiving plants (Betti et al., 2021). To further investigate whether miRNAs are delivered between different plant individuals, overexpressing miR399/miR156 lines and wild-type were cultured in the same liquid medium. The results showed that a higher concentration of miR399/miR156 was detected in the medium, and the target genes in wild-type plants were down-regulated (**Figure 2B**), suggesting that miRNAs were secreted outside of plants and functioned in their neighbors (Betti et al., 2021). This study demonstrated that miRNAs function as novel signaling molecules in plant-to-plant communication.

### 3.2 Cross-kingdom regulation of miRNAs in plant-microorganism interaction

Apart from miRNA communication within and across plant species, miRNAs also transfer between plants and their microbial pathogens. Cross-kingdom miRNA regulation between plants and fungal pathogens is bidirectional (Wang et al., 2016; Huang et al., 2019a). One way that microbial pathogens alter the expression of host genes involved in immunity is by delivering small RNAs into host plants (Wang et al., 2016; Huang et al., 2019a). For instance, the gray mold *Botrytis cinerea* causes gene silencing in different host plant species by introducing a variety of small RNAs into plant cells (Weiberg et al., 2013). The miRNA-like RNA 1 from *Puccinia striiformis* (Pst) was found to inhibit gene expression in host wheat (*Triticum aestivum*), reducing the host plants’ resistance to Pst (Wang et al., 2017a). However, the miRNAs from plant hosts may also be reciprocally transported into pathogens to silence genes linked to virulence (Wang et al., 2016; Cai et al., 2018b; Huang et al., 2019a). In response to pathogen *Verticilium dahlia*, cotton (*Gossypium hirsutum*) miR159 and miR166 were transferred into fungal hyphae, where they silenced the virulence genes (Zhang et al., 2016c). Another study showed that *Arabidopsis*-derived miR166 was transferred to fungal pathogen *B. cinerea.* to inhibit pathogen gene expression (Cai et al., 2018b). Since these plant miRNAs are delivered into microbial pathogens and efficiently silence the virulence-related genes, they can be applied to protect crops. Several major crops, including barley, wheat, and soybean, have been genetically engineered to produce these cross-kingdom plant miRNAs to regulate pathogenic diseases caused by viruses, viroids, fungi, oomycetes, and bacteria (Niu et al., 2021). Additionally, it has been proven that some pathogenic microorganisms can absorb external small RNAs from their surroundings (Wang et al., 2016; Cai et al., 2018a), making the direct application of small RNA spay to protect plants against the microbial pathogens. For example, it has been found that rice can be effectively protected against blast disease by spaying small RNAs that target *Magnaporthe oryzae MoDES1* gene (Sarkar and Roy-Barman, 2021). Similarly, the application of exogenous small RNAs confers plant protection against the pathogens *B. cinerea* and *Sclerotinia sclerotiorum* (McLoughlin et al., 2018). Therefore, the transboundary regulation of miRNAs between plants and microorganisms inspires the development and application of innovative methods for crop disease control in agriculture (Niu et al., 2021; Qiao et al., 2021).

### 3.3 Cross-kingdom regulation of miRNAs between plants-insects

**Figure 3:**
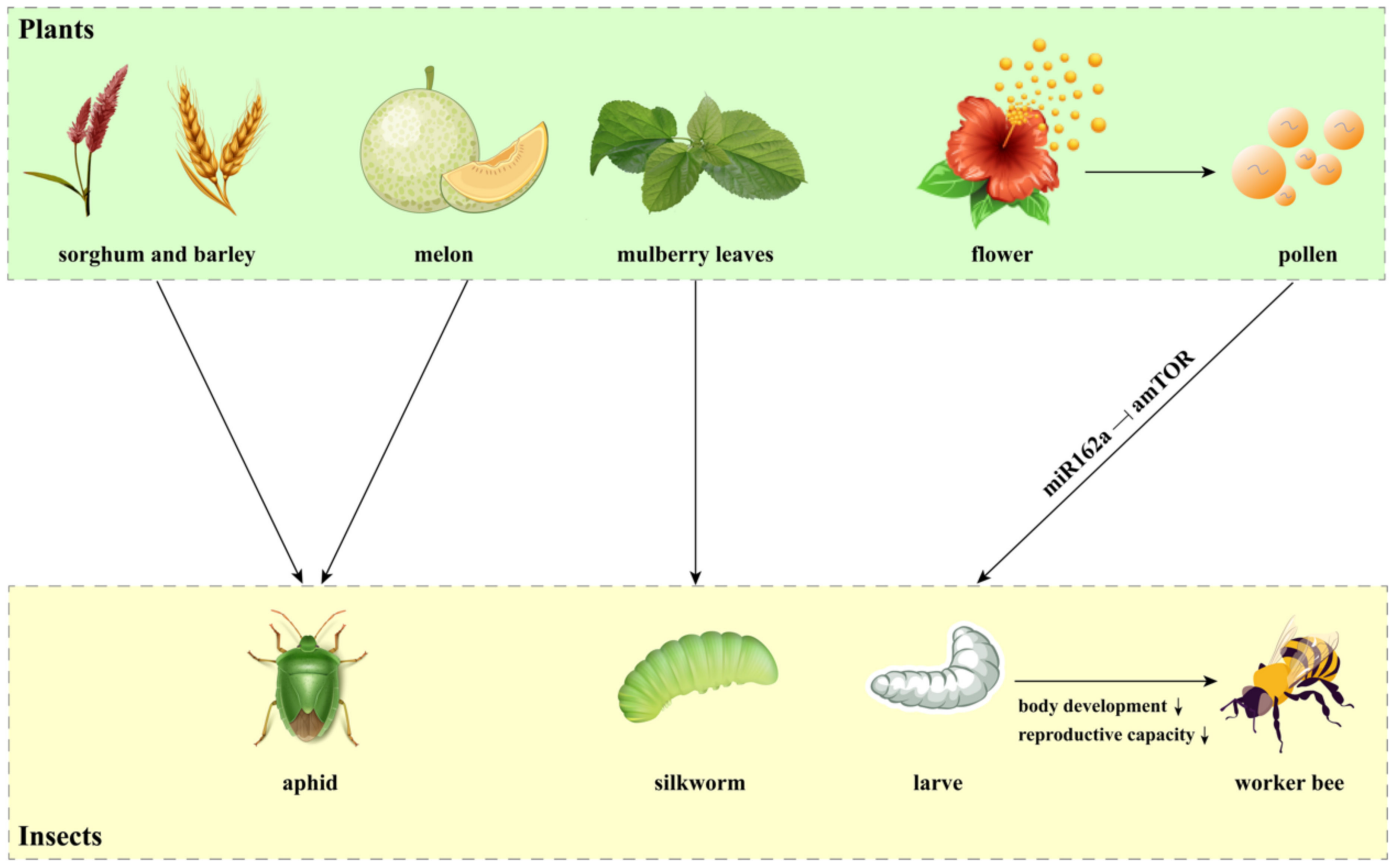
Cross-kingdom miRNAs in plant-insect interactions. This figure illustrates how miRNAs derived from specific plants are transferred and act as regulatory molecules in the bodies of herbivorous insects. Notably, miRNAs from sorghum, barley, and melon have been detected in various aphid species, while miRNAs from mulberry leaves have been identified in silkworms. Additionally, pollen-derived miR162a plays a key role in the regulatory process of honey bee caste formation by targeting amTOR gene.

The cross-kingdom transmission of miRNAs has also been demonstrated in plant-insect interactions (Chi et al., 2023). Some herbivorous insects utilize foliar, root, or phloem resources as their food, which facilitate the transport of host plant miRNAs into their bodies (**Figure 3**). Several cereal miRNAs, including 13 miRNAs from *Sorghum bicolor* and 3 miRNAs from *Hordeum vulgare*, have been found in two grain aphid species (*Schizaphis graminum* and *Sipha flava*), indicating that plant miRNAs are transferred into the herbivorous insects (Wang et al., 2017b). Plant miRNAs have also been identified in the bodies of cotton-melon aphid *Aphis gossypii* during feeding on melon phloem sap (Sattar et al., 2012). Similarly, mulberry (*Morus alba*) miRNAs were discovered in silkworm hemolymph and multiple tissues after feeding on mulberry leaves, confirming that plant miRNAs are taken up by insects orally and transported into various tissues (Jia et al., 2015). Intriguingly, plant miRNAs are involved in transboundary regulation through plant-pollinator interactions. An excellent example illustrating the pollen-derived miRNA regulatory process is honey bee caste formation (Masood et al., 2016; Zhu et al., 2017). Plant miRNAs have been found to delay development, decrease body weight and length, and reduce the reproductive capacity of honeybees, thus promoting worker bee formation (Zhu et al., 2017). Mechanically, pollen miR162a inhibits the expression of the amTOR gene in bees, which determines caste development (**Figure 3**) (Zhu et al., 2017).

### 3.4 The roles of miRNAs in cross-boundary regulation between plants-mammals

Now the transboundary regulation of miRNAs from plants to mammals is the focus of attention, as the plant-derived miRNAs may be used as novel agents to improve human health (Li et al., 2023; Xu et al., 2024b). As early as 2012, it was discovered that rice-derived miR168a could target the mRNA of murine low-density lipoprotein receptor adapter protein 1 (LDLRAP1), reducing its protein level in the liver (Zhang et al., 2012). This was the first scientific proof of the transboundary regulation by plant miRNAs in mammals, implying that mammals can take up functional miRNAs from dietary plant food. In 2015, it was discovered that honeysuckle (*Lonicera japonica*) miR2911 could be consumed by mice and directly targeted to influenza A virus (IAV) (Zhou et al., 2015). Honeysuckle miR2911 effectively inhibited IAV’s *PB2* and *NS1* gene expression *in vivo* and *in vitro* (Zhou et al., 2015). This research proved that the plant-derived miRNAs suppressed viral proliferation in mammals, which suggested the promising application of plant miRNAs as antiviral drugs. Later, plant miR159 was identified in human bodies and recognized for its potential to inhibit breast tumor cell growth by targeting the transcription factor 7 (TCF7). TCF7 is important for tumor initiation by mediating Wnt/β-catenin signaling (Chin et al., 2016). Similarly, miR156a was found to be enriched in broccoli and the synthetic miR156a mimics inhibited epithelial-mesenchymal transition (EMT) in human nasopharyngeal carcinoma (NPC) cells by specifically targeting the 3’ UTR of the junctional adhesion molecular A (JAM-A) *in vitro* (Tian et al., 2016). Plant miR159a and miR156c from nut exhibit anti-inflammatory properties via suppressing TNF superfamily member 1a (Tnfrsf1a) expression in macrophages and adipocytes (Aquilano et al., 2019). In 2020, clinical data showed that honeysuckle-derived miR2911 can prevent the replication of SARS-CoV-2, accelerating recovery from COVID-19 infection (Zhou et al., 2020). Ginger-derived miRNA (aly-miR396a-5p) was reported to decrease SARS-CoV-2-induced lung inflammation in mice by repressing the expression of the *Nsp12* gene (Teng et al., 2021). Additionally, the plant miR171 was found to modulate the mTOR pathway by targeting G protein subunit alpha 12 (GNA12) (Gismondi et al., 2021). The plant miR167e-5p has the ability to influence adipogenesis via negative regulation of β-catenin in 3T3-L1 cells (Chen et al., 2022b). Garlic-derived miRNA (Han-miR3630-5p) can bind to and inhibit toll-like receptor 4 (TLR4), thus alleviating DSS-induced colitis (Zhu et al., 2023b). Previous work in our lab showed that honeysuckle-derived miR2911 inhibits HPV-positive cervical cancer cell proliferation via directly targeting the oncogenes of HPV E6 and E7 (Chi et al., 2024). So far, examples of cross-kingdom gene regulation of plant miRNAs are still being discovered. The following table summarizes the existing examples of transboundary regulation of plant miRNAs in mammals (**Table 1**).

**Table 1:** Identified plant miRNAs involved in transboundary regulation of disease-related genes.

miRNAs	Plant source	Interacting organism	Target gene	Function	Year	References
miR168a	*Oryza sativa*	Human/Mouse	LDLRAP1	An increase in plasma LDL and cholesterol levels	2012	(Zhang et al., 2012)
miR172	*Brassica oleracea*	Mouse	No available	No available	2014	(Liang et al., 2014)
miR2911	*Lonicera japonica*	Mouse	PB2/NS1	Inhibition of influenza A virus (IAV) replication	2015	(Zhou et al., 2015)
plant miRNAs (such as miR156a, miR168a, miR390a, miR528)	Watermelon juice and mixed fruits	Human	No available	No available	2015	(Liang et al., 2015)
miR159	*Arabidopsis thaliana, Glycine max, Brassica oleracea*	Mouse	TCF7	Inhibit proliferation of breast cancer	2016	(Chin et al., 2016)
miR168	*Fragaria vesca*	Human	Toll-like receptor 3 (TLR3)	Modify dendritic cell ability to respond to inflammatory agents	2016	(Cavalieri et al., 2016)
miR166a/miR159	*Brassica campestris*	Mouse	No available	No available	2016	(Chen et al., 2016)
miR156a	*Brassica oleracea*	Human	JAM-A	Inhibits EMT of NPC cells	2016	(Tian et al., 2016)
miR2910	*Populus euphratica*	Human	SPRY4	No available	2017	(Liu et al., 2017)
miR14	*Curcuma longa*	Human	No available	Improve rheumatoid arthritis	2017	(Sharma et al., 2017)
Cac-miR-4723–3p	*Camptotheca acuminata*	Human	DLG2	No available	2017	(Kumar et al., 2017)
zma-miR164a-5p	*Zea mays*	Pig	CSPG4/OTX1/PLAGL2	No available	2017	(Luo et al., 2017)
miR156a	*Brassica oleracea/Spinacia oleracea/Lactuca sativa*	Human	JAM-A	Reduce monocyte adhesion induced by inflammatory cytokines	2018	(Hou et al., 2018)
miR5338	Rape bee pollen	Rat	Mfn1	Treatment of benign prostatic hyperplasia (BPH)	2018	(Chen et al., 2018)
miR2911	*Lonicera japonica*	Mouse	VP1	Inhibition of Enterovirus 71 (EV71) replication	2018	(Li et al., 2018a)
mdo-miR7267-3p	*Zingiber officinale*	Mouse	YcnE	Improve intestinal barrier function and ameliorate mouse colitis	2018	(Teng et al., 2018)
miR2911	*Lonicera japonica*	Mouse	IE62	Inhibition of Varicella-zoster virus (VZV) replication	2019	(Huang et al., 2019b)
miR167e-5p	*Astragalus membranaceus*	Human	β-catenin	Inhibition of intestinal cell proliferation	2019	(Li et al., 2019)
HJT-sRNA-m7	*Rhodiola rosea*	Mouse	α-smooth muscle actin (α-SMA)/fibronectin/collagen type III α 1 (COL3A1)	Anti-pulmonary fibrosis	2019	(Du et al., 2019)
miR156c miR159a	Dried nuts	Mouse	Tnfrsf1a	Decrease Tnfrsf1a protein and affect TNF-α in adipocytes	2019	(Aquilano et al., 2019)
miR2911	*Lonicera japonica*	Human	No available	Suppress SARS-CoV-2 replication	2020	(Zhou et al., 2020)
miR01 miR02	*Gastrodia elata Blume*	Human	A20	Involve in cell cycle, immune regulation	2020	(Xia et al., 2020)
miR2911	*Lonicera japonica*	Human	TGFβ1	Inhibit colon tumor growth	2021	(Liu et al., 2021)
miR4057	Manuka trees (honey)	Mouse	NLRP3 inflammasome	Alleviate inflammation and liver damage	2021	(Chen et al., 2021b)
gma-miR-159a	Soybean	Mouse	GSK-3β	Prevent hepatic fibrosis	2021	(Yu et al., 2021)
miR171 miR396a-5p miR168-3p	Many plants such as *Malus domestica, Oryza sativa* *Zingiber officinale* *Oryza sativa* *Hordeum vulgare*	Human Rat Human	GNA12 Nsp12 Mitochondrial complex I related genes	Modulate mTOR pathway in HEK293 cells Suppress lung inflammation caused by SARS-CoV-2 Prevent glucose transporter 1 (GLUT1)-related dysfunctions	2021 2021 2022	(Gismondi et al., 2021) (Teng et al., 2021; Akao et al., 2022)
Bol-miR172a	*Brassica oleracea*	Human	FAN	Anti-inflammatory effect	2022	(Kasarello et al., 2022)
miR167e-5p	*Astragalus membranaceus*	Human	β-catenin	Promote 3T3-L1 adipocyte adipogenesis	2022	(Chen et al., 2022b)
miR-CM1	*Phellinus linteus*	Human/ Mouse	Mical2	Inhibit ultraviolet-induced skin aging	2022	(Han et al., 2022)
miR159	*Brassica oleracea* var. *italica*	Mouse	PhaZ2/celC/rnY in bacteria	Regulate gut microbiota	2023	(Xu et al., 2023)
osa−miR172d−5p	*Oryza sativa*	mouse	Tab1	Ameliorate lung fibrosis	2023	(Kumazoe et al., 2023)
miR858a/miR858b miR166a-3p	*Houttuynia* *cordata*	Human	NP in H1N1 ORF1ab in SARS-CoV-2	Against respiratory RNA viruses	2023	(Zhu et al., 2023a)
miR-7972	*Rehmannia glutinosa*	Mouse	GPR161	Alleviate LPS-induced acute lung injury	2023	(Qiu et al., 2023)
Gas-miR36-5p	*Gastrodia elata*	Human	GSK-3	Neuroprotective effects	2023	(Lu et al., 2023)
Han-miR3630-5p	*Allium sativum*	Mouse	TLR4	Inhibit TLR4 expression and reduce the pro-inflammatory cytokines	2023	(Zhu et al., 2023b)
miR2911	*Lonicera japonica*	Human	E6/E7	Inhibit cervical cancer cell proliferation by targeting HPV E6/E7	2024	(Chi et al., 2024)
peu-MIR2916-p3	*Allium sativum*	Mouse	No available	Ameliorate murine colitis	2024	(Wang et al., 2024b)
miR156	*Panax ginseng*	Mouse	No available	Regulate energy metabolism and immunity	2024	(Wang et al., 2024a)
miR168a	*Oryza sativa*	Mouse	No available	Attenuates dextran sulfate sodium-induced colitis	2024	(Xu et al., 2024a)

## 4 The plant miRNAs in extracellular vesicles

Exosomes, which store bioactive chemicals such as proteins and RNA molecules, have been well characterized in mammalian cells. Recently, plant miRNAs have been discovered to mediate communication within the plant kingdom and cross kingdoms by being stably packaged into plant-derived exosome-like nanoparticles (PENs) (Zhang et al., 2016b; Chen et al., 2022a; Cieslik et al., 2022; Urzi et al., 2022). PENs can be extracted from various plants and serve as nanocarriers for the delivery of biological cargo, including plant-derived miRNAs. For instance, a study identified a range of miRNAs in edible PENs from 11 different species, with numbers ranging from 32 to 127 (Xiao et al., 2018). PENs from grapes have been shown to contain 96 miRNAs (Ju et al., 2013), while ginger-derived PENs contain 125 distinct miRNAs (Zhang et al., 2016a). Approximately 398 miRNAs have been identified in PENs from ginseng (Xu et al., 2021). Several studies have demonstrated that PENs exert biological effects mediated by bioactive miRNAs (Xiao et al., 2018; Potesta et al., 2020; Kalarikkal and Sundaram, 2021; Teng et al., 2021; Xu et al., 2021). The mechanism by which plant miRNAs selectively load into PENs has been discovered (He et al., 2021). Appropriate small RNA packaging requires the involvement of RNA binding proteins, such as AGO1, RNA helicases, and annexins (He et al., 2021). The PENs share structural and functional similarities with mammal exosomes (Cai et al., 2021; Teng et al., 2021). The phospholipid bilayer and surface polysaccharides of PENs protect the encapsulated cargo from degradation in the gastrointestinal tract, resulting in higher stability and bioavailability of the miRNAs (Yang et al., 2018; Kalarikkal and Sundaram, 2021). Notably, the miRNAs packed in the PENs are highly resistant to RNase and tolerant to both acidic and alkaline environments (Wang et al., 2020; Umezu et al., 2021; Qin et al., 2022), enabling the transfer of plant miRNAs through the digestive tract after oral intake.

## 5 PEN-mediated absorption and delivery

**Figure 4:**
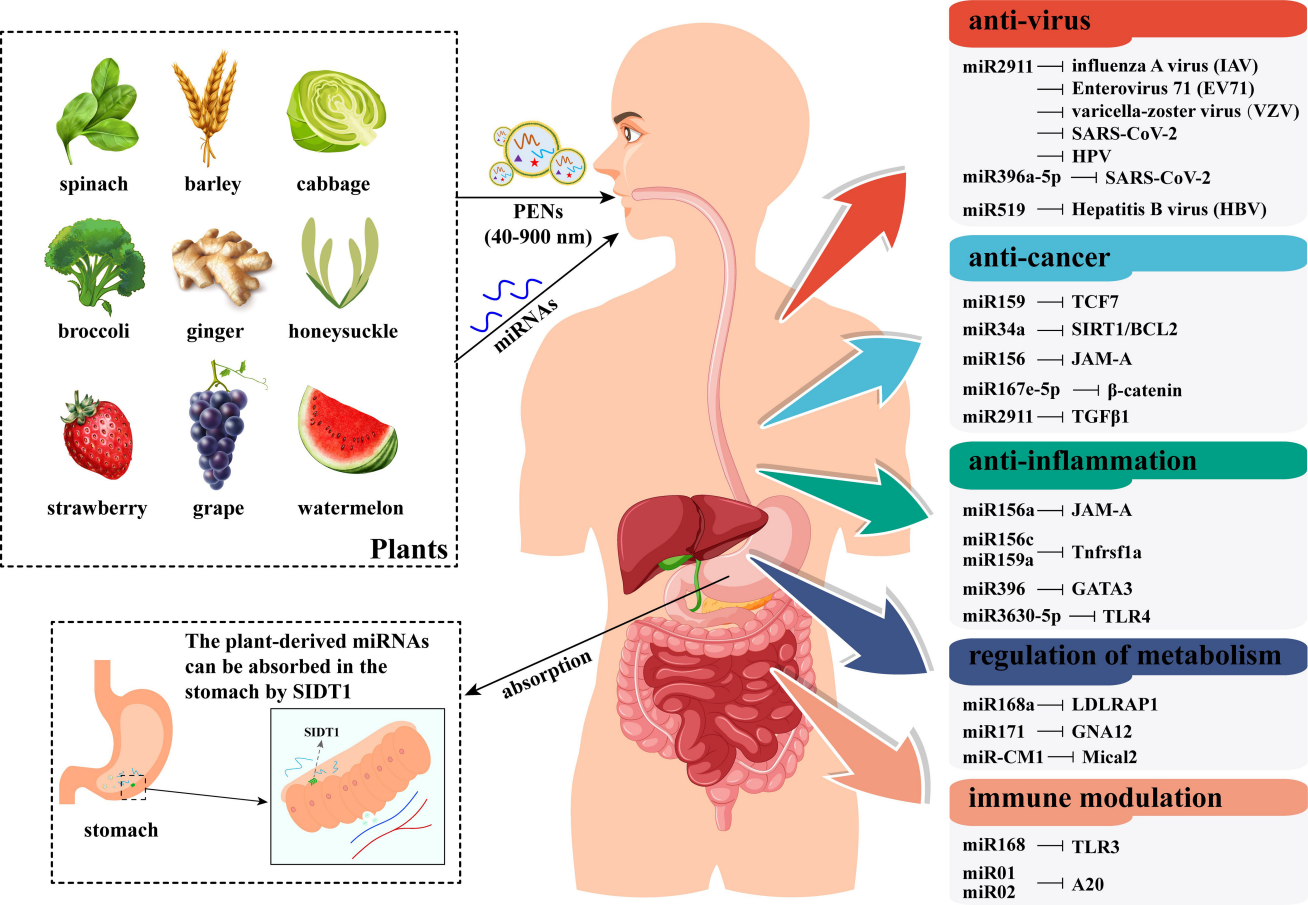
A schematic model of plant miRNAs’ transport, uptake, and effects in human bodies. This figure illustrates the process by which plant miRNAs (either naked or encapsulated in PENs) are obtained orally through diet. After passing through the gastrointestinal tract, plant miRNAs are absorbed by the SIDT1 protein in the stomach. These absorbed miRNAs are transported to target organs and cells via the circulatory system. Ultimately, plant miRNAs regulate disease-related gene expression in a cross-kingdom manner, acting as novel active substances that benefit human health. The information in the right boxes lists plant miRNAs and their primary targets in humans.

The PENs can be absorbed by intestinal macrophages via micropinocytosis and clathrin-dependent endocytosis (Wang et al., 2014; Umezu et al., 2021). Importantly, a recent study confirmed that plant miRNAs in PENs are more easily absorbed by mammals (Teng et al., 2018). A study indicated that miRNAs from vegetables or fruits are generally absorbed in the gastric mucosal cells of the stomach (Chen et al., 2021a). In detail, the SID1 transmembrane family member 1 (SIDT1), which is rich in the stomach, functions as an essential transporter that facilitates the absorption of dietary miRNAs into these cells (**Figure 4**) (Chen et al., 2021a). Besides, it is worth noting that the uptake of mature plant miRNA by SIDT1 was significantly increased under acidic culture conditions (Chen et al., 2021a). Following absorption, the plant miRNAs enter the bloodstream and circulate to various tissues such as the stomach, intestine, lung, liver, and bladder, where they modulate target genes in recipient mammalian cells (**Figure 4**) (Zhang and Hong, 2019; Chen et al., 2021a). In particular, the gas-miR36-5p from *Gastrodia elata* was found to prevent Alzheimer’s disease in a mouse model through targeted inhibition of GSK-3 activity, suggesting plant miRNAs can cross the blood-brain barrier and treat central nervous system diseases (Lu et al., 2023).

In addition to oral uptake, PENs can also be administered via other routes, including intranasal administration, intraperitoneal administration, intravenous administration, and transdermal administration (Feng et al., 2023). This suggests that cargo miRNAs can reach target organs or cells through multiple administration methods. Thus, plant-derived miRNAs may represent a promising therapeutic strategy against various diseases, including viral infections, tumors, inflammation, metabolic disorders, and immune modulation (**Figure 4**). The transportation and administration of plant miRNAs in mammals are then achieved through a PEN-mediated mode. In fact, PENs have been used to deliver exogenous small RNAs to disrupt gene expression of multiple diseases. For instance, grapefruit–derived PENs were deployed to deliver miR17 to mouse brain tumor via intranasal route (Zhuang et al., 2016). Similarly, ginger-derived PENs were able to transfer exogenous small RNAs to intestinal epithelial tissue (Zhang et al., 2017) or subcutaneous tumor cells (Li et al., 2018b). Recently, PENs from four foods have been shown to serve as effective nanocarriers for exogenous miRNAs (Lopez de Las Hazas et al., 2023). The result suggested that PENs can enhance the biological stability of exogenous miRNAs against RNase degradation and ferry miRNAs toward cellular uptake (Lopez de Las Hazas et al., 2023). Furthermore, it was found that host plants transferred miRNAs to the fungal pathogen *Botrytis cinerea* primarily through PENs (Cai et al., 2018b). Therefore, PENs may serve as vehicles for delivering plant miRNAs from donor plants to other recipient organisms.

## 6 Conclusions and perspectives

Growing evidence suggests that plant miRNAs may serve as an ‘RNA language’ to interact various organisms, including plants, microorganisms, insects, and mammals. Despite the increasing knowledge about cross-kingdom regulation of plant miRNAs, many questions remain unanswered. For instance, how are miRNAs secreted from donor plants and absorbed by recipient plants during interspecies transfer? Which types of plant miRNAs—those associated with AGO proteins, miRNA duplexes, or mature single strands—are mobile across species? Additionally, do plant miRNAs enter receiving plants via transporter proteins similar to SID-1 in nematodes (Shih and Hunter, 2011) or SIDT1 in mammals (Chen et al., 2021a)? While evidence suggests that plant miRNAs are effectively encapsulated and transported via PENs, how do these miRNAs retain functionality in different organisms during cross-kingdom regulation? This is particularly important given that most plant miRNAs are 3’ methylated, which differs from animal miRNAs, and mammalian AGO proteins generally do not bind to methylated small RNAs. Moreover, the process by which plant miRNAs are packaged into PENs requires further clarification. Several studies indicate that absorbed plant miRNAs are packaged into extracellular vesicles in mammalian circulation (Zhang et al., 2012; Luo et al., 2017; Chen et al., 2021a). However, it remains unclear whether these enclosed plant miRNAs are repackaged into mammalian exosomes in the intestine or retained in their original PENs. Addressing these questions through further experimentation will significantly enhance our understanding of RNA biology and facilitate practical applications.

Pathogenic fungi and pests pose serious threats to crop production. Given the transboundary regulation of plant miRNAs in combating these threats, plant miRNAs could be valuable tools for crop protection against diseases (Mahanty et al., 2023). The host-induced gene silencing (HIGS) strategy involves genetically engineered plants producing small RNAs to target virulence-related genes, proving effective against various fungal pathogens and pests (Qi et al., 2019). However, HIGS raises concerns regarding transgenic implementations in agriculture. To address this, spray-induced gene silencing (SIGS) has been developed, where RNAs are directly applied to plant leaves. Using stable nanovesicles, RNA sprays conjugated with nanoparticles can enhance the efficacy of pathogen gene silencing (Qiao et al., 2021). Nevertheless, SIGS faces challenges related to RNA uptake efficiency and production costs.

As research uncovers the roles of plant miRNAs in regulating disease-related genes in mammals, their potential applications in therapeutic interventions—particularly in anti-cancer and anti-viral contexts—are becoming increasingly evident (Zhang et al., 2023). Numerous miRNAs have been identified as tumor suppressors or oncogenic in human contexts (Slack and Chinnaiyan, 2019), leading to a burgeoning field of miRNA therapeutics against cancer (Rupaimoole and Slack, 2017; Diener et al., 2022). As highlighted in **Table 1** and **Figure 4**, several plant miRNAs influence cancer cell cycles, proliferation, and apoptosis, suggesting their potential as anti-cancer agents. Interestingly, unusual 1-nt shorter miRNA isoforms are prevalent in cancers, and ectopic expression of plant RDR1 can inhibit cancer cell proliferation by modifying defective miRNAs (Qi et al., 2022), indicating shared editing processes of small RNAs between plants and animals.

On the anti-viral front, plant miRNAs have garnered attention for their efficacy against various viruses, including honeysuckle miR2911, which targets influenza A, varicella-zoster virus, and enterovirus 71. Notably, during the COVID-19 pandemic, honeysuckle-derived miR2911 was found to prevent the replication of SARS-CoV-2, promoting recovery from infection. Additionally, ginger-derived aly-miR396a-5p and rlcv-miR-rL1-28-3p have shown potential in inhibiting SARS-CoV-2 gene expression (Teng et al., 2021). Houttuynia cordata-derived miRNAs have also been validated for targeting respiratory RNA viruses, such as miR858a and miR858b against H1N1 and miR166a-3p against SARS-CoV-2 (Zhu et al., 2023a). Recently, the plant beverage Amrtan Ocean 2911, rich in honeysuckle miR2911, has been approved for market entry. These advancements suggest significant translational medicine applications for plant miRNA-based bioengineering.

Moreover, the role of plant miRNAs may extend to herbal medicine. Historically, research on active pharmacological components in herbal medicine has focused on secondary metabolites. However, with the recognition of transboundary roles, herb-derived miRNAs could emerge as novel active ingredients. A recent study established the Bencao small RNA Atlas by sequencing small RNA libraries from 265 traditional Chinese medicines, identifying 21,757 miRNAs, of which 17,509 (80.4%) were predicted to target at least one human gene. Furthermore, 39.1% of human genes were predicted to be targeted by these herbal miRNAs (Cao et al., 2023).

Despite the promising potential of plant miRNAs, their inherent risks must be carefully considered. Due to conserved sequences, a single plant miRNA may target a broad spectrum of genes in mammals, potentially impacting entire cellular pathways even with moderate effects on individual targets. Off-target effects also warrant attention. Thus, comprehensive analyses of target genes for individual plant miRNAs are essential. Proper dosages and administration strategies should be tailored to specific tissues and cell types. Finally, multidimensional validation of the effects of plant miRNAs on cells and animals must be conducted prior to clinical trials to mitigate unforeseen interactions.
